# Apple Procyanidins Suppress Amyloid **β**-Protein Aggregation

**DOI:** 10.1155/2011/784698

**Published:** 2011-08-02

**Authors:** Toshihiko Toda, Tadahiro Sunagawa, Tomomasa Kanda, Motoyuki Tagashira, Takuji Shirasawa, Takahiko Shimizu

**Affiliations:** ^1^Molecular Gerontology, Tokyo Metropolitan Institute of Gerontology, 35-2 Sakae-cho, Itabashi-ku, Tokyo 173-0015, Japan; ^2^JAC Co., Ltd., 1-2-7 Higashiyama, Meguro-ku, Tokyo 153-0043, Japan; ^3^Asahi Breweries, Ltd., 1-1-21 Midori, Moriya-shi, Ibaraki 302-0106, Japan; ^4^Ageing Control Medicine, Juntendo University Graduate School of Medicine, 3-1-3-10-201 Hongo, Bunkyo-ku, Tokyo 113-0033, Japan

## Abstract

Procyanidins (PCs) are major components of the apple polyphenols (APs). We previously reported that treatment with PC extended the mean lifespan of *Caenorhabditis elegans* (Sunagawa et al., 2011). In order to estimate the neuroprotective effects of PC, we investigated the antiaggregative activity of PC on amyloid **β**-protein (A**β**) aggregation, which is a pathological hallmark of Alzheimer's disease. We herein report that PC significantly suppressed A**β**42 aggregation and dissociated A**β**42 aggregates in a dose-dependent manner, indicating that PC is a potent suppressor of A**β** aggregation. Furthermore, PC significantly inhibited A**β**42 neurotoxicity and stimulated proliferation in PC-12 cells. These results suggested that the PC and AP acted as neuroprotective factors against toxic A**β** aggregates.

## 1. Introduction

Polyphenols are comprised of several groups of compounds (e.g., anthocyanins, flavonols, and phenolic acids) and belong to a family of plant secondary metabolites that widely accumulate in plants as well as fruits [1]. The polyphenols extracted from apples (*Malus pumila* Mill., Rosaceae) mainly contain procyanidins (PCs), as well as known proanthocyanidins, leukocyanidins, and condensed tannins, which account for approximately 65% of apple polyphenols (AP) [2]. PC is formed by catechin oligomers composed of (−)-epicatechin and (+)-catechin monomers (MNs) [3]. PC is also found in a variety of fruits, berries, and several medicinal plants or plant components, such as grape (*Vitis vinifera*) seeds [4], bilberry (*Vaccinium myrtillus*) [5], hawthorn (*Crataegus monogyna*) [6], ginkgo (*Ginkgo biloba*) [7], tormentil (genus* Potentilla*) [8], and oak (genus* Quercus*) [9]. 

It has been reported that several polyphenols including PC show potential benefits to human health, such as antioxidant [10], antitumor [11], anti-inflammatory effects [12], and longevity [13], as well as protective effects on glucose consumption [14]. We have also revealed that apple PC showed antiallergy [15], antitumor [16], and antiobesity effects [17] in a rodent model and longevity effects on *Caenorhabditis elegans* [2]. In this context, PC might be a promising polyphenol that can prevent age-related diseases. 

Alzheimer's disease (AD) is a typical age-related and progressive neurodegenerative disease with memory impairment in later life. AD is diagnosed by amyloid accumulation, which is observed as a deposition in the hippocampus and cerebral cortex, named a senile plaque, composed of amyloid *β*-proteins (A*β*) [18]. A*β*42, which consists of 42-residues, is observed mainly in the core of senile plaques. The protein forms strong aggregates themselves that are associated with neurotoxicity *in vitro * [19]. Several polyphenols attenuate insoluble A*β* accumulation [20–22]. 

This study addressed the anti-neurodegenerative effects of PC and AP by investigating whether PC can prevent the aggregation of A*β*. The results demonstrated that apple PCs suppress A*β* aggregation and cytotoxicity *in vitro* and strongly contribute to neuroprotection in AP. 

## 2. Materials and Methods

### 2.1. Compounds

The APs were prepared from immature apples (*Malus pumila* Mill. cv. Fuji), and the methods employed for AP preparation were described previously [2]. The PCs (procyanidins, catechin oligomers) accounted for 63.8% of the AP, and the methods used to prepare the PC and monomer fractions were used as described previously [2]. The A*β*42 peptide was purchased from the Peptide Institute. 

### 2.2. Thioflavin-T Fluorescence Assay

The aggregative abilities of A*β*42 were evaluated using the thioflavin-T (Th-T) method as described previously [22]. Various concentrations of test samples were coincubated with A*β*42 (final concentration 20 *μ*M) for 6 to 48-hour at 37°C in 50 mM phosphate buffer (pH 7.4) containing 100 mM NaCl. In the case of posttreatment, A*β*42 (final concentration 20 *μ*M) was preincubated without PC for 48 hours at 37°C, and then various concentrations of polyphenols were added and incubated for 0.5 to 5 hours at room temperature. The incubated samples were stored at −80°C until measurement. Th-T (final concentration 5 *μ*M, Sigma-Aldrich) in 50 mM glycine-NaOH buffer (pH 8.5) was added to the samples and incubated for 30 minutes at room temperature. The measurements were performed on a SPECTRA max GEMINI XS fluorescence microplate reader (Molecular Devices). Fluorescence intensity was measured using 442 nm for excitation and 485 nm for emission. The percentage of A*β* aggregate inhibition was calculated by comparing the fluorescence values of the test samples with those of vehicle solutions with A*β*42. 

### 2.3. Separation of A*β* Precipitation

Polyphenol samples (100 *μ*g/mL AP, 65 *μ*g/mL PC, and 35 *μ*g/mL MN) were coincubated with A*β*42 (final concentration 20 *μ*M) for 24 hours as described before [22]. A 300 *μ*L aliquot of the reacted samples was centrifuged at 20,000 ×g for 30 minutes at 4°C. The A*β* pellets were dried at room temperature before optical photographs were obtained using an SZX9 microscope (12.5-fold, OLYMPUS). The pellets were resolved in 6 M guanidine-HCl (pH 4.5–7.5, Sigma-Aldrich) and diluted by 15-fold RIPA buffer containing 0.1% sodium dodecyl sulfate (SDS, Sigma-Aldrich). A*β* concentrations were measured using a DC protein assay kit (Bio-Rad Laboratories). The supernatants of the sedimented samples were denatured at 85°C for 2 minutes with EzApply solution (ATTO) containing 1% SDS and 50 mM dithiothreitol before separation by electrophoresis at 25 mA on 20% polyacrylamide gels in Tris-glycine buffer (SDS-PAGE). Soluble A*β* peptides in 5 *μ*L of reacted solutions were detected using Coomassie brilliant blue staining (Quick-CBB, Wako Pure Chemical) according to the manufacturer's protocol.

### 2.4. Estimation of Cell Viability

PC-12 cells (RCB0009, RIKEN BioResource Center) were used as a neural cell model in order to evaluate the cytotoxicity of A*β* peptides [23]. The experimental procedure was a previously described method [22]. Briefly, after the incubation of the PC-12 culture (2 × 10^4^ cells per well) for 16 hours at 37°C, the cells were pretreated for 1 hour with or without various concentrations of filter-sterilized polyphenols, followed by treatment with 1 *μ*M A*β*42 for an additional 36 hours. Then, cells were treated with thiazolyl blue tetrazolium bromide (MTT, final concentration 0.5 mg/mL, Sigma-Aldrich) for 4 hours at 37°C. After solubilization with SDS (Sigma-Aldrich), the rate of formazan formation was evaluated by measuring the absorbance at 570 nm using a VersaMax microplate reader (Molecular Devices). Data are given as percentages of the control values without PC and A*β*42. 

### 2.5. Statistical Analyses

All data were presented as the means ± s.e.m. The StatMate III software package was used for all statistical analyses. Differences were analyzed by Student's *t*-test, and multiple comparisons between groups were performed with Dunnett's test for posthoc analysis. *P* values of <0.05 or <0.001 were considered to be statistically significant. 

## 3. Results

### 3.1. In Vitro Anti-A*β* Aggregative Effects of Procyanidins

**Figure 1:**
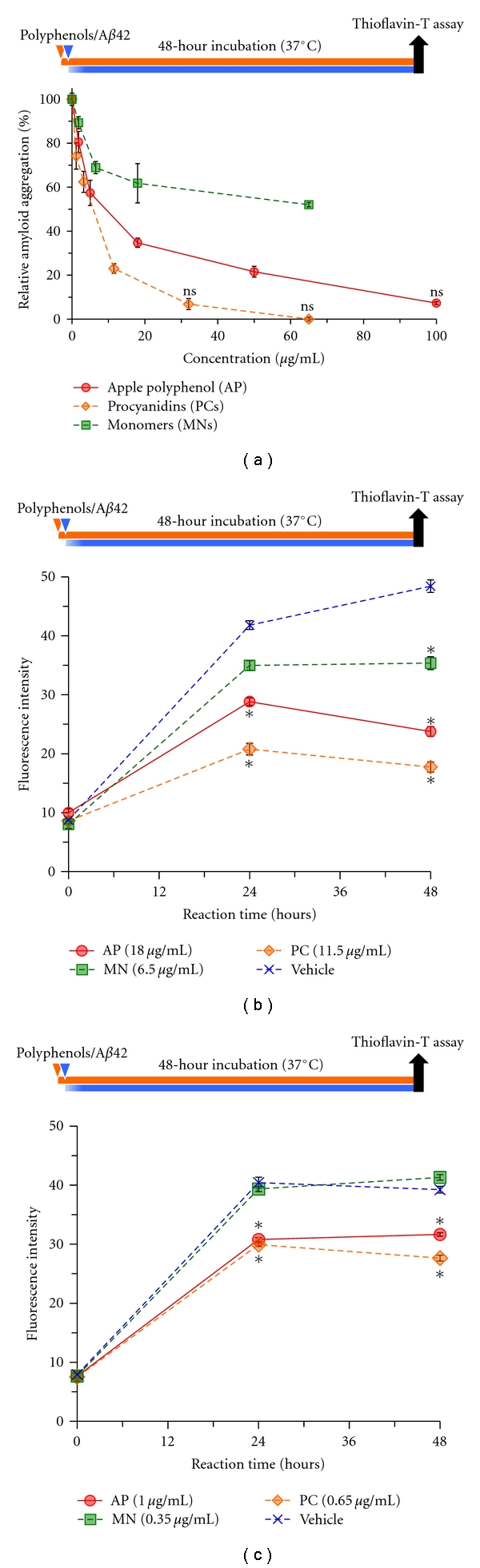
Procyanidins suppress A*β* aggregation in vitro. (a) Dose-dependent suppression of A**β**42 aggregation by polyphenols as indicated by thioflavin-T (Th-T) analysis. Various concentrations of apple polyphenol (AP), procyanidins (PC), and monomers (MN) were incubated with A**β**42 (final concentration 20 *μ*M) for 48 hours. (Differences from 0 hour (baseline), ns: not significant (*P* < 0.01)). (b, c) Time-dependent A**β**42 aggregation in the presence of polyphenols as indicated by the Th-T assay. (b) A*β*42 (20 *μ*M) was incubated with AP (18.0 *μ*g/mL), PC (11.5 *μ*g/mL), and MN (6.5 *μ*g/mL). (c) A**β**42 (20 *μ*M) was incubated with AP (1.0 *μ*g/mL), PC (0.65 *μ*g/mL), and MN (0.35 *μ*g/mL). (differences compared to vehicle groups, *P < 0.001 (Dunnett's test)). Values are the means ± s.e.m., *n* = 5.

To examine the effect of PC on A*β* aggregation, we performed thioflavin-T (Th-T) fluorescence assays for A*β*42 (Figure 1). The PC comprised approximately 65% of the AP, and the remaining approximately 35% was regarded as the monomer fraction (MN) [2]. In the absence of PC, A*β*42 (20 *μ*M) formed Th-T-binding aggregates 48 hours after incubation, whereas the Th-T fluorescence intensity was dramatically decreased in a dose-dependent manner by AP and PC (Figure 1(a)). In addition, 100 *μ*g/mL AP and 32.5 *μ*g/mL PC completely abrogated A*β* aggregation throughout the incubation period (Figure 1(a), ns; not significant compared with 0 hour, *P* < 0.01), while MN resulted in a limited decrease in the A*β* aggregation (Figure 1(a)). Since 32.5 *μ*g/mL PC corresponded to 100 *μ*g/mL AP, this result suggests that PCs have approximately 2-fold the anti-A*β* aggregative ability compared to AP. Interestingly, a bioavailable dose of PC (11.5 *μ*g/mL), which had been detected in rat plasma after the oral administration of 1,000 mg AP/kg body weight [24], significantly suppressed A*β* aggregation (Figure 1(b)). Furthermore, low-dose AP and PC (1.0 and 0.65 *μ*g/mL, resp.) significantly suppressed A*β* aggregation, while 0.35 *μ*g/mL MN did not suppress the aggregation at any time during the incubation period (Figure 1(c)). These results suggest that apple PC effectively suppressed A*β* aggregation compared to MN, including epicatechins and catechins.

**Figure 2:**
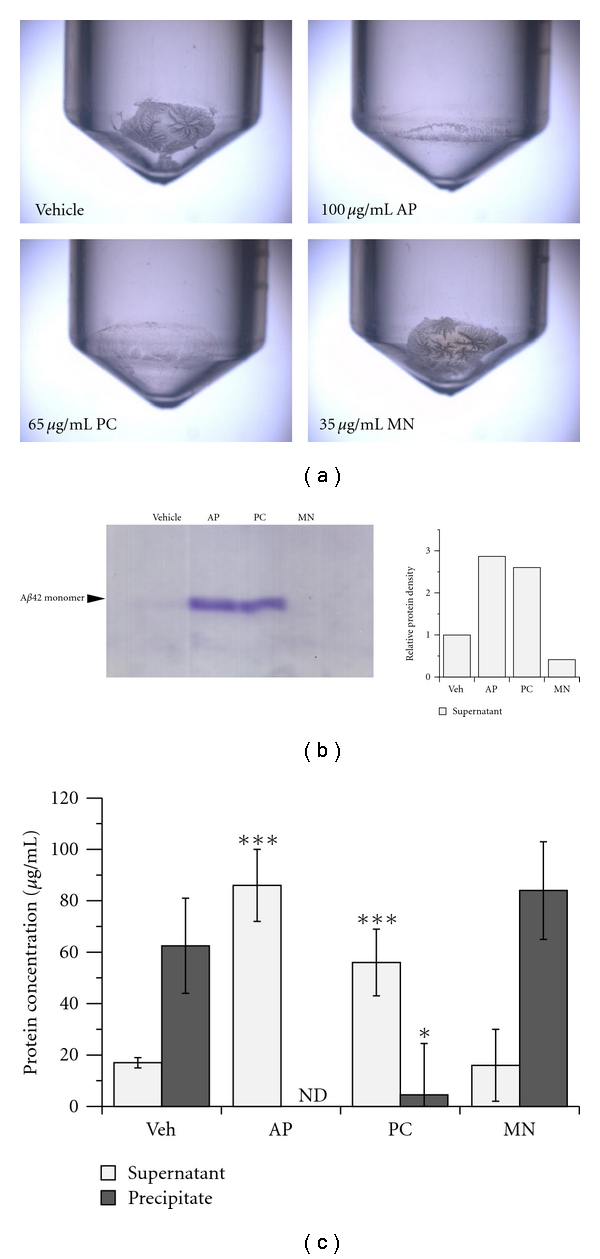
Procyanidins extinguish A*β* precipitation. (a) Microscopic observation of A*β*42 precipitates by centrifugation in the presence of polyphenols. The aggregates were observed by 12.5-fold magnification. (b) SDS-PAGE analysis of soluble A*β*42 peptide of supernatants in reaction mixtures. Arbitrary density of monomeric A*β*42 bands was calculated in the right column, 5 *μ*L/lane. (c) A*β* concentrations of supernatant and precipitate in the reaction mixtures. AP (100 *μ*g/mL), PC (65 *μ*g/mL), and MN (35 *μ*g/mL) were incubated with A*β*42 (20 *μ*M) for 24 hours and centrifuged to fractionate supernatants and pellets as described in Section 2 (ND: not detected; differences compared to vehicle groups, ***P < 0.001 and *P < 0.05 (Student's *t*-test)). Values are the means ± s.e.m., *n* = 3.

We next analyzed the inhibitory effect of PC on amyloid aggregation using a centrifugation method, to exclude the possibility that exogenous compounds affected Th-T fluorescence intensity [25]. We observed the typical aggregates of A*β* (20 *μ*M) after 48 hours incubation (Figure 2(a); vehicle). Treatment with 100 *μ*g/mL AP and 65 *μ*g/mL PC completely diminished the aggregates, while 35 *μ*g/mL MN treatment did not (Figure 2(a)). We also detected monomeric A*β*42 in the supernatant by SDS-PAGE following 100 *μ*g/mL AP and 65 *μ*g/mL PC treatment (Figure 2(b)). A protein concentration assay also demonstrated that 100 *μ*g/mL AP and 65 *μ*g/mL PC markedly inhibited A*β* aggregation (Figure 2(c)). Taken together, PC and AP exhibited strong anti-amyloidogenic ability *in vitro*. 

### 3.2. Dissociative Activity of Procyanidins against A*β* Aggregates

**Figure 3:**
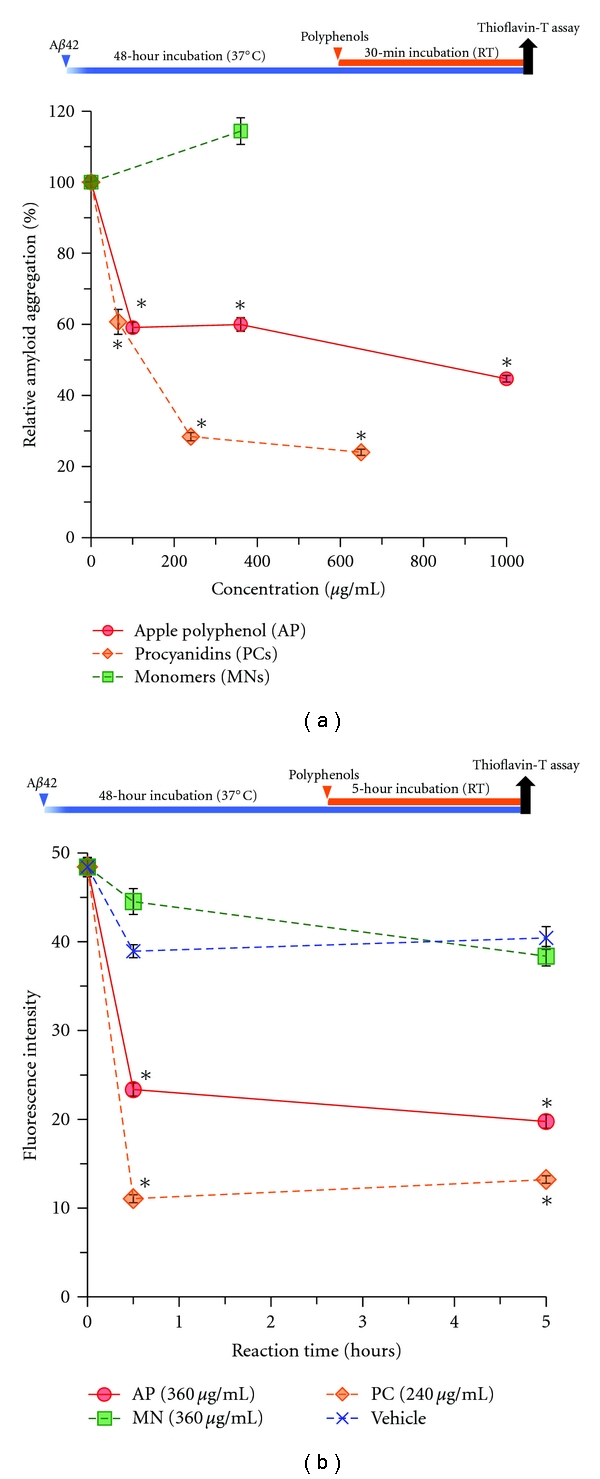
Procyanidins dissolve A*β* aggregates in vitro. (a) Dose-dependent dissociation of A*β*42 aggregates by polyphenols determined using Th-T analysis. Samples were preincubated with A*β*42 for 48 hours (final concentration 20 *μ*M) and then incubated with various concentrations of AP, PC, or MN for an additional 30 minutes. (b) Time-dependent A*β*42 disaggregation in the presence of polyphenols determined by the Th-T assay. Samples were preincubated with A*β*42 (20 *μ*M) for 48 hours and then incubated with AP (360 *μ*g/mL), PC (240 *μ*g/mL), or MN (360 *μ*g/mL). (Compared to vehicle groups, *P < 0.001 (Dunnett's test)). Values are the means ± s.e.m., *n* = 3.

To investigate whether the PC can dissolve aggregated A*β*42, we also performed the Th-T assay using a posttreatment protocol. After the incubation of A*β*42 (20 *μ*M) for 48 hours, various concentrations of AP, PC, and MN were added and then were additionally incubated for 30 minutes (Figure 3(a)). PC effectively dissociated A*β*42 aggregates in a dose-dependent manner (65–650 *μ*g/mL), while AP resulted in limited dissociation (Figure 3(a)). On the other hand, A*β* disaggregation was not observed after 360 *μ*g/mL MN treatment (Figures 3(a) and 3(b)). In addition, AP and PC also significantly dissociated A*β* aggregates at 30 minutes, and the dissociation continued until 5 hours after addition of the compounds (Figure 3(b)). In contrast, AP and PC (50 *μ*g/mL and 32.5 *μ*g/mL, resp.) failed to dissociate A*β* aggregates (data not shown). This result indicated that AP and PC dissociated A*β* aggregates in a high-concentration manner. 

### 3.3. Neuroprotective Effects of Procyanidins against A*β*42-Induced Toxicity

**Figure 4:**
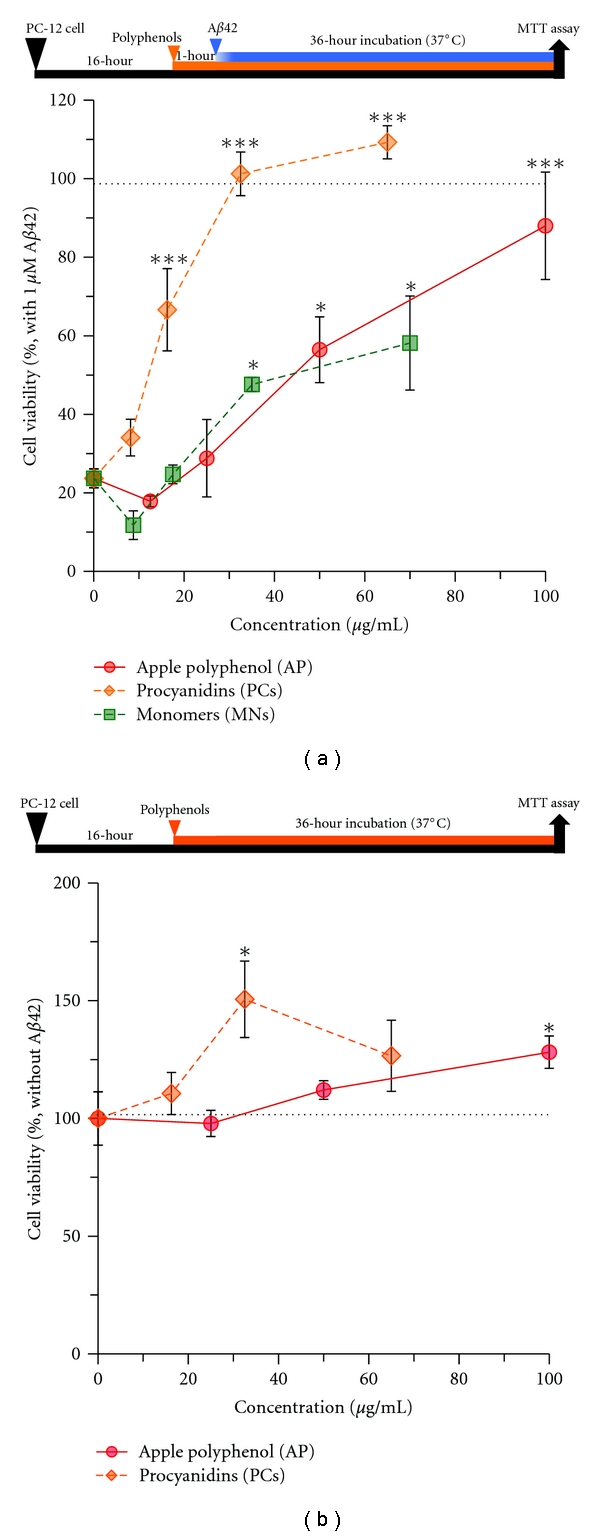
Procyanidins restore viability of PC-12 cells treated with A*β*42. Cells were pretreated with various concentrations of AP, PC and MN for 1 hour, and then, incubated with (a) or without (b) 1 *μ*M A*β*42 for 36 hours. Cell viability was measured by the MTT assay. Values are the means ± s.e.m., *n* = 3. (Compared to the control group (0 *μ*g/mL), ***P < 0.001 and *P < 0.05 (Dunnett's test)).

A*β*42 plays a pivotal role in the pathogenesis AD because of its potent aggregative ability and neurotoxicity [26]. PC-12 cells were established to measure cellular viability associated with A*β*42 treatment. These cells were used to investigate the neuroprotective effect of PC against A*β*42-induced neurotoxicity using an MTT assay [27]. The A*β*42 (1 *μ*M) induced cytotoxicity (23.7 ± 2.4% viability) in the cells after coincubation for 36 hours (Figure 4(a)). When PC-12 cells were preincubated with AP or PC for 1 hour and then treated with A*β*42 for 36 hours, AP and PC significantly inhibited cytotoxicity in a dose-dependent manner. In particular, 32.5 *μ*g/mL PC restored cell viability to 101.2 ± 5.6% (Figure 4(a)). On the other hand, 100 *μ*g/mL AP restored cell viability to 88.0 ± 13.7% (Figure 4(a)). When the cells were treated with AP or PC alone (without A*β*42) for 36 hours, a beneficial effect on cell viability was observed at concentrations of 32.5 *μ*g/mL PC and 100 *μ*g/mL AP (Figure 4(b)). These results indicated that apple PC prevented A*β*42-induced cytotoxicity on PC-12 cells. 

## 4. Discussion

Polyphenols extracted from functional plants have been reported to show antiaggregative and anti-neurotoxic properties *in vitro* [28], and it is not clear whether AP has these functions. Polyphenols, however, usually have poor bioavailability, and this is even more marked for macromolecular substances containing PC [29, 30]. On the other hand, the long-term administration of several polyphenols effectively prevents AD-like pathologies and memory impairment in a mouse model of AD [20, 31, 32]. We have also shown that orally silymarin treatment, extracted from milk thistle (*Silybum marianum*), attenuates AD-like phenotypes in a mouse model of AD [22]. Furthermore, a specific Porter method and high-performance liquid chromatography/tandem mass spectrometry identified apple PC oligomers at a concentration of 11.4 *μ*g/mL in rat plasma 2 hours after single intake of high dose 1,000 mg AP/kg body weight [24]. The present study showed that PC inhibited A*β* aggregation and neurotoxicity at an IC_50_ of 4.8 *μ*g/mL and 9.4 *μ*g/mL, respectively (Figures 1 and 4), suggesting that apple PC is able to prevent amyloidogenesis at least in vessels. Therefore, it might be able to ameliorate AD-like pathologies and memory impairment in a mouse model of AD receiving long-term administration of PC. Further studies are needed to clarify this matter.

In Figure 1, Th-T analyses on A*β* aggregation indicated that the IC_90_ of AP was 87.8 *μ*g/mL, while the IC_90_ of PC was 24.7 *μ*g/mL, which corresponded to 38.0 *μ*g/mL AP (Figure 1(a)). Consequently, the inhibitory activity of PC against A*β* aggregation was approximately 2-fold higher than that of AP. Furthermore, the dissociative effect of AP on A*β* was saturated at concentration of 100 *μ*g/mL, while that of PC continued to increase until 240 *μ*g/mL (Figure 3(a)). These results corresponded to cell viability of MTT assays (Figure 4), suggesting that PCs have a more potent ability to promote A*β* disaggregation and neuroprotection than whole APs. In contrast, MN showed limited depression of A*β* aggregation. Whole AP and MN may contain factors that counteract the antiaggregative and neuroprotective activity of PC. 

Hirohata et al. reported that myricetin, an antioxidative polyphenol, exerted an anti-amyloidogenic effect by reversible binding to the A*β* fibril structure *in vitro* [33]. Furthermore, Kirschner et al. reported that curcumin suppresses A*β* aggregation via hydrogen binding to A*β*42 at Glu^11^-Gly^25^ [34]. Kumar et al. also reported a structural analysis demonstrating that curcumin directly binds to Gln^15^, Glu^22^, and Asp^23^ of A*β*42 [35]. The Gln^15^ to Ile^32^ region is predicted to be an intramolecular *β*-sheet in A*β* assemblies; thus curcumin can disturb A*β* assembly due to binding to A*βin vitro *as well as *in vivo* [20, 26]. Apple PC binding to A*β* at position Gln^15^-Ile^32^ might directly inhibit the conformation of A*β*42. Pasinetti et al. reported that grape seed polyphenolic extract including PC interferes with paired helical filament formation by direct physical intercalation with tau molecules [36]. Therefore, apple PC might have a similar suppressive effect on intermolecular aggregation of tau as well as A*β*42. Interestingly, we also observed a brown-colored pellet that formed following treatment of A*β*42 aggregates with PC and AP (data not shown), suggesting that PC could bind to A*β*42 aggregates. On the other hand, treatments with neither vitamin-C (2 mM) nor the potent antioxidative agent, EUK-134 (40 *μ*M), did not suppress the A*β* aggregation (data not shown), thus suggesting that the antiaggregative activity of PC might likely be independent of its antioxidant properties. Although Yatin et al. reported that vitamin-E (VE) does not suppress A*β*42 aggregation [37], Yang et al. revealed that *α*-tocopherol quinone derived from VE inhibits A*β* aggregation in a dose-dependent manner [38]. These findings suggest that the chemical structure of these compounds impacts the inhibition of A*β* aggregation rather than their antioxidative capacity. 

In Figure 4(a), PC showed potent neuroprotective effects in a dose-dependent fashion, demonstrating that PC can play a role in both A*β*-disaggregation and cellular survival. In addition, the neuroprotective effect of PC was stronger than that of AP as well as MN (Figure 4(a)), which was consistent with the antiaggregative activity of PC (Figures 1 and 3), suggesting that PC suppress A*β* aggregation leading to neuroprotection against A*β*42. Interestingly, we also found an additional effect of PC on cell viability analysis. When PC-12 cells were treated with PC (32.5 *μ*g/mL) without A*β*, the percentages of cell viability were enhanced to over 100% (Figure 4(b)). Furthermore, AP also significantly induced cellular viability at a concentration of 100 *μ*g/mL (Figure 4(b)). Miura et al. previously reported that treatment with low-dose PC enhanced cell proliferation, while high-dose PC induced apoptosis in melanoma cells [16]. Recently, Choi et al. reported that PC treatment inhibits endogenous histone acetyltransferase, subsequently suppresses cell proliferation, and increases cell death in prostate cancer cells [39]. These results suggest that PC can regulate cell proliferation in a dose-dependent manner. Therefore, apple PC might not only suppress A*β* aggregation but also modify neuronal cell proliferation, thus contributing to the neuroprotective effect against A*β*-induced cytotoxicity. 

In conclusion, apple PC acted as a potent suppresser of abnormal A*β* aggregation and a showed protective effect on neuronal survival *in vitro*. Since apple PC is a safe and an inexpensive food factor, it might be promising for long-term treatment. Our study suggests a novel activity of apple PC, further supporting the consideration of their use for either the prevention or treatment of A*β* aggregation associated with neurodegenerative disorders.
